# Peripheral and central auditory impairment linked to heavy metal exposure in Nicaraguan children from artisanal mining communities

**DOI:** 10.7189/jogh.16.04192

**Published:** 2026-07-17

**Authors:** Marissa Kachadoorian, Torri Lee, Jessica Fitzgerald, Michaela Geffert, Catherine Rieke, Odile Clavier, Adrian Fuente, Jiang Gui, Jie Zhou, Siting Li, Brian Jackson, Margaret Karagas, Karen Mojica, Marvin González-Quiroz, Christopher Niemczak, Jay Buckey, James Saunders

**Affiliations:** 1 Geisel School of Medicine at Dartmouth College, Hanover, New Hampshire, USA; 2 Department of Surgery – Section of Otolaryngology, Dartmouth Hitchcock Medical Center, Lebanon, New Hampshire, USA; 3 School of Speech Pathology and Audiology, University of Montreal, Montreal, Canada; 4 Creare LLC, Hanover, New Hampshire, USA; 5 Department of Otolaryngology, Vivian Pellas Hospital, Managua, Nicaragua; 6 Department of Earth and Planetary Sciences, Dartmouth College, Hanover, New Hampshire, USA; 7 Department of Environmental and Occupational Health, Kate Marmion School of Public Health, The University of Texas at San Antonio, San Antonio, Texas, USA; 8 Centre for Kidney and Bladder Health, University College London, London, UK

**Keywords:** central auditory processing, artisanal and small-scale gold mining, ototoxicity, heavy metal exposure, dichotic digits test, mercury

## Abstract

**Background:**

Children living in artisanal and small-scale gold mining (ASGM) communities are exposed to numerous metals with possible ototoxic and neurotoxic effects. Tests of peripheral and central auditory function can reflect toxicity to the ear and brain, respectively. This study investigated the link between metal exposure and peripheral and central auditory processing (CAP) in children exposed to metals in ASGM settings.

**Methods:**

In a cross-sectional study, 211 children aged 6–18 in ASGM communities performed audiometric (pure-tone audiometry, PTA) and CAP assessments (dichotic digits test, DDT). Toenail samples were analysed for 21 metals. Data on noise exposure and other covariates were collected via questionnaire. Linear and Elastic Net regression models were used to identify predictors of auditory impairment.

**Results:**

Median metal concentrations except chromium (Cr) and selenium (Se) exceeded published reference values. For CAP assessments, several metals were identified by Elastic Net (lead (Pb), antimony (Sb), uranium (U), vanadium (V)) and linear regression (aluminium (Al), cadmium (Cd), cobalt (Co), Cr, copper (Cu), iron (Fe), mercury (Hg), manganese (Mn), strontium (Sr), thallium (Tl), U, V), respectively. Both methods identified U and V as potential predictors. For peripheral auditory function, Elastic Net selected silver (Ag), Cd, Cr, Hg, and molybdenum (Mo), while linear regression identified Ag, arsenic (As), Co, Cr, Cu, Hg, Mn, Mo, Pb, Sr, and Tl. Silver (Ag), Cr, Hg, and Mo were consistently selected by both methods. At high-frequency peripheral thresholds, Elastic Net identified As, nickel (Ni), Pb, and tin (Sn), and linear regression identified Ag, Al, As, Cd, Co, Cu, Fe, Hg, Mn, Mo, Pb, and V. Both analyses selected As and Pb as potential predictors.

**Conclusions:**

CAP and peripheral auditory test performance correlated with metals known to have ototoxic and neurotoxic effects, as well as metals with previously unrecognised neurotoxic potential. These findings support measuring multiple metals in future epidemiologic studies to examine potential metal toxicities beyond those with known neurotoxicity.

Heavy metal and metalloid exposure is common in many low- and middle-income countries (LMICs), leading to deleterious health effects, including kidney damage, neurotoxicity, and neurocognitive impairment [1]. This is particularly concerning in artisanal and small-scale gold mining (ASGM) communities in LMICs, where inadequate resources and infrastructure hinder identifying and mitigating the exposures [2]. Artisanal and small-scale gold, mining operations employ approximately 15 million people worldwide, including 4–5 million women and children, often in remote and impoverished regions [3]. Mercury (Hg) is commonly used in ASGM to extract gold making it the largest anthropogenic source of Hg contamination globally [4]. Mercury toxicity poses a significant global health risk, contributing an estimated 1.22 to 2.39 million disability-adjusted life years (DALYs) – a burden comparable to that of Parkinson Disease [5]. In addition to Hg, residents of ASGM communities are frequently exposed to high levels of other potentially toxic heavy metals [6]. For example, lead (Pb), arsenic (As), and cadmium (Cd) are also known to have nephrotoxic, neurotoxic and ototoxic effects [7,8]. The toxicological profiles of many other metals remain poorly understood, underscoring the need for further research in these vulnerable populations.

Early neurotoxic effects can be subtle and challenging to detect or monitor. One potential way to detect these changes is through abnormalities in central auditory processing (CAP). While peripheral hearing primarily depends on the cochlea to detect and transmit auditory information, CAP occurs at a higher level, within the brainstem and cortex. Our research shows that CAP test performance correlates with and predicts neurocognitive impairments across various domains, including processing speed, memory, and executive function [9–11]. In one recent study, performance on central auditory tests predicted future neurocognitive function in at risk paediatric populations [12]. Central auditory processing deficits can result from changes in various auditory functions, including temporal processing, pattern recognition, speech-in-noise perception, dichotic listening, and sound localisation [13].

Our team has developed a tablet-based audiometric and CAP test battery, available in both English and Spanish, which can be administered by minimally trained community health workers. This system is built on an established and validated platform for pure-tone audiometry that integrates a highly noise-attenuating headset, the Wireless Automated Hearing Test System (WAHTS) [14,15,16], with a tablet-based user interface and data management application (TabSINT) [17]. This tablet-based system was deployed to explore the relationship between toenail concentrations of 21 heavy metals and performance on peripheral pure-tone audiometry, and CAP tests in children living in ASGM communities. Toenail clippings were selected as an easily transportable and stable sample of long-term metal exposure in this setting. Questionnaires were used to collect data on exposure to noise and ototoxins. The primary goal was to establish whether heavy metal concentrations were elevated in this population and to explore potential correlations between these concentrations and auditory test performance. Currently, the effects of heavy metal exposure on hearing thresholds and CAP remain poorly understood [7]. A better understanding of these relationships may reveal early signs of neurotoxicity, expand our understanding of vulnerabilities with the auditory pathway, and may potentially lead to the development of non-invasive biomarkers in at-risk populations.

## METHODS

### Subject recruitment

All test procedures were conducted with the approval of the Nicaraguan Ministry of Health and the institutional review boards of the National Autonomous University of Nicaragua at León (UNAN-Leon), Dartmouth-Hitchcock Medical Center, and Dartmouth College. Participants were children and adolescents aged 6–18 years recruited from Santo Domingo and La Libertad communities from June 2022 to September 2022 and engaged in active ASGM operations in the Chontales region of Nicaragua. The study size was determined by the number of eligible patients meeting inclusion criteria during the study period. Eligibility criteria included having a family member working in ASGM or personal involvement in mining operations. Children with active ear infections, known middle ear pathologies, or severe cognitive impairments that would preclude completion of the tests were excluded from the study. Informed consent in Spanish was obtained for all subjects. Subjects over 15 years of age were allowed to consent for themselves according to the legal jurisdictions of Nicaragua. Subjects under 15 years of age provided verbal and written assent when age appropriate. Given the cultural circumstances of the research, parental consent from a single parent was considered sufficient for this study. All forms received approval by the UNAN-Leon IRB. All procedures were conducted in compliance with the STROBE checklist recommendations for observational studies. The completed checklist is provided in the **Online Supplementary Document**.

### Audiometric Testing Equipment and Software

Otoscopy using a Firefly video otoscope and tympanometry with an Interacoustics MT10 middle-ear analyzer were performed on all participants to rule out middle ear pathology. If conductive hearing loss could not be ruled out based on these tests, those subjects were excluded from statistical analyses.

Cross-sectional paediatric auditory assessments were performed using the WAHTS (Edare LLC, Lebanon, NH), a wireless headset with noise-attenuation equivalent to a single-walled sound booth, enabling high-quality audiometric testing in remote ASGM communities [14]. A tablet-based mobile CAP evaluation (mCAPE) test battery was developed in Spanish for use with the WAHTS, including the Dichotic Digits Test (DDT) reported in this study. Further details, including results and feasibility of mCAPE test battery employing community health workers (CHWs) are available elsewhere [18]. Data collection was facilitated by TabSINT, an open-source software framework for audiometric assessments and general-purpose questionnaires. The data were stored locally on the tablet and later uploaded to Gitlab, an internet-based repository, for analysis once Wi-Fi connection became available [16,17]. Audiometric results, along with detailed socioeconomic and risk factor data, were managed using the Research Electronic Data Capture (REDCap) database [19,20].

### Community Health Worker Training

Three CHWs were locally trained to support study activities through a two-day intensive training session that combined classroom instruction and hands-on practice. The training covered human subject protection (CITI certification), and a range of study procedures, including standard Pure Tone Average (PTA) testing, administration of the mCAPE test battery, tympanometry, and otoscopy. Additional training included online modules from the International Hearing Care Technician Certification programme, toenail sampling techniques, the REDCap platform for administrating tablet-based questionnaires, and the TabSINT software for audiometrics.

### Demographic/Risk Factor Assessment Questionnaire

A comprehensive 70-item questionnaire was administered to gather data on demographics, noise exposure, personal or familial mining activity, neurological symptoms, ototoxic medication use, and environmental exposure to ototoxic chemicals/heavy metals. Environmental factors included water sources, household fumigation, dietary habits, and pesticide exposure. Self-reported sex assigned at birth was collected. Noise exposure questions covered both recreational and work exposure (primary exposure questions), as well as whether the person had experienced symptoms from noise exposure (tinnitus, hearing loss, pain). Participants were classified as having primary exposure (yes to ≥1 primary noise exposure question), severe exposure (yes to ≥1 primary and ≥1 symptom question), or no exposure (no to all primary questions). These categorical groups were then used as a covariate in the statistical analysis examining the relationships between heavy metal concentrations and audiometric or CAP test results.

### Peripheral and Central Auditory Processing Procedures

Our laboratory has previously demonstrated the feasibility of audiometric testing and the mCAPE test battery in Nicaraguan children [6,18]. A brief overview of two key components of this test battery is provided here.

### Pure Tone Audiometry

Pure tone audiometry thresholds were determined using a modified Hughson-Westlake method (0.5–8.0 kHz, 12 total thresholds). A binaural PTA was calculated based on the six frequencies tested for each participant.

### Dichotic Digits Test

The Dichotic Digit Test (DDT) was used to evaluate binaural integration of sound by presenting two numbers in each ear simultaneously (four total) [21]. The DDT was scored as the overall percentage of the four correctly identified numbers (20 sequences of four-digit presentations, total 80 digits). Participants with PTA asymmetry of 10 dB or greater between ears were excluded from this analysis (n = 5). To aid comprehension, participants watched a video training module prior to completing the DDT.

### Toenail sample collection and heavy metal concentration analysis

Toenail clippings were collected from each participant as biomarkers of long-term heavy metal exposure [22]. Samples were stored and shipped in metal-free envelopes and shipped to Trace Elements Analysis Laboratory at Dartmouth College for analysis of metal concentrations using inductively coupled plasma mass spectrometry (ICP-MS). Prior to analysis, nail clippings were washed to remove external contamination and digested using a microwave digestion system. Quality control procedures adhered to US Environmental Protection Agency (EPA) guidelines outlined in EPA 6020. Detailed methodology is described elsewhere [6]. The concentrations of the 21 metals and metalloids were analysed in the biological tissue, including arsenic (As), mercury (Hg), lead (Pb), aluminium (Al), manganese (Mn), silver (Ag), cadmium (Cd), cobalt (Co), Chromium (Cr), copper (Cu), iron (Fe), molybdenum (Mo), nickel (Ni), antimony (Sb), selenium (Se), tin (Sn), strontium (Sr), titanium (Ti), uranium (U), vanadium (V), and zinc (Zn). For this study, these metals and metalloids will be referred to as ‘heavy metals’. The concentration of index metals (Se and Zn) in this cohort are comparable to those from other international populations suggesting a limited effect of contamination.

### Statistical analysis

All audiometric thresholds, tympanometry data, and otoscopy results were reviewed for validity. Data were analysed using Elastic Net penalized regression, which is well suited for situations with multiple correlated predictors. This method was used to screen for features with potential predictive relevance. Three Elastic Net regressions were performed, with DDT performance, PTA, and hearing thresholds at 8kHz as the outcomes. The 21 metal concentrations served as predictors. Z-scores were created for all metal variables using the entire data, and these were used in the analysis as predictors. To address the variability in age, time working in the mines, noise exposure *etc*., the Elastic Net regression was conducted using 1000 bootstrapped samples of the data set using resampling with replacement. This step generated new populations for analysis that varied in distribution of age, hearing loss, noise exposure, and other confounders. The number of times a metal was included as a predictor was counted. Metals that were included as predictors more that 50% of the time were considered as possible significant predictors.

Multiple linear regression were performed using individual log-transformed toenail metal concentrations as predictor variables and audiometric thresholds and CAP test results as outcomes. This statistical method was applied in parallel to the Elastic Net regression, offering more conventional effect estimates for comparison. Age and categorical noise groups (primary, severe, or no exposure) were included as confounders in all models. Statistical analyses were performed in *R* version 4.4.0 (R Project for Statistical Computing, Vienna, Austria), MATLAB (The Mathworks Inc., Natick, Massachusetts, USA), and StatPlus (AnalystSoft Inc., Brandon, Florida, USA). To control the expected proportion of false positives among all statistically significant results, a False Discovery Rate (FDR) [23] threshold of 0.2 was applied to the linear regression results, providing a balanced approach to multiple-comparison correction that is particularly well-suited for large data sets with innumerable potential correlations. Findings were considered statistically significant if they met both a *P*-value threshold of 0.05 and the FDR-adjusted threshold of <0.2. Fourteen additional covariates, including sex, cyanide exposure, pesticide exposure, head injury, premature birth, fumigation chemical exposure, family history of congenital hearing loss, recurrent severe ear pain, otitis media, neonatal hospitalisation, and use of ototoxic medications (gentamicin, amikacin) were evaluated for their influence on the relationship between metal concentration and auditory outcomes. These covariates were considered significant if their inclusion altered the *P*-value of metal-outcome relationship beyond 0.05. Metal concentrations were compared against reference medians proposed by Goullé et. al. [24] and, for Fe, against a proposed maximum value described by Meramat et al. [25].

## RESULTS

A total of 211 Nicaraguan children and adolescents were recruited for the study. Nine participants were excluded, four due to incomplete data and five due to middle ear pathology, resulting in a final sample of 202 participants. Ages ranged from six to 18 (mean age ± standard deviation (SD) = 12 ± 2.8 years) with a slightly higher proportion of males (59%) (**Table 1**). Most participants (85%) reported active involvement in ASGM operations, working an average of 32 hours per week. Additionally, participants reported frequently being near noisy equipment (52 hours per week on average).

**Table 1:** Demographics, exposure information, peripheral and central auditory test results

Characteristics	Value*
**Sample, n**	209
**Gender**	
Female	86 (41)
Male	123 (59)
**Age in years, x̄ (SD)**	12 (2.8)
**Number of participants working in ASGM**	178 (85)
**Hours worked weekly, x̄ (SD)**	32 (24)
**Time spent with noisy machinery per week, hours, x̄ (SD)**	52 (45)
**Peripheral hearing tests**	
Binaural PTA (dB), x̄ (SD)	11 (4.0)
**Central Auditory Processing Tests**	
DDT (%), x̄ (SD)	81 (3.0)
**Neurologic symptoms**	
Frequent headaches	131 (63)
Memory loss	103 (49)
Problems with dizziness and/or balance	60 (29)
Tinnitus	58 (28)
Difficulty understanding speech but can hear sounds normally	50 (24)
Difficulty holding a conversation when there is background noise	
*Usually*	35 (17)
*Sometimes*	79 (38)
*Rarely*	66 (32)
*Never*	28 (13)
Tremors	32 (15)
Paresthesia	25 (12)

ASGM – artisanal small scale gold mining, DDT – dichotic digits test, PTA – pure tone audiometry, x̄ – mean, SD – standard deviation

*Values are presented as n (%) unless otherwise specified.

High rates of neurological symptoms were observed among participants. Frequent headaches were reported by 63%, memory loss by 49%, and tremors by 15%. Additional symptoms included dizziness and/or balance problems (29%), difficulty understanding speech despite normal hearing (24%), challenges holding conversations in noisy environments (17%), paresthesias (12%), and unusual changes in skin or nail colour (11%). Notably, 15 children (7%) had a diagnosis of chronic kidney disease.

### Metal levels

**Figure 1:**
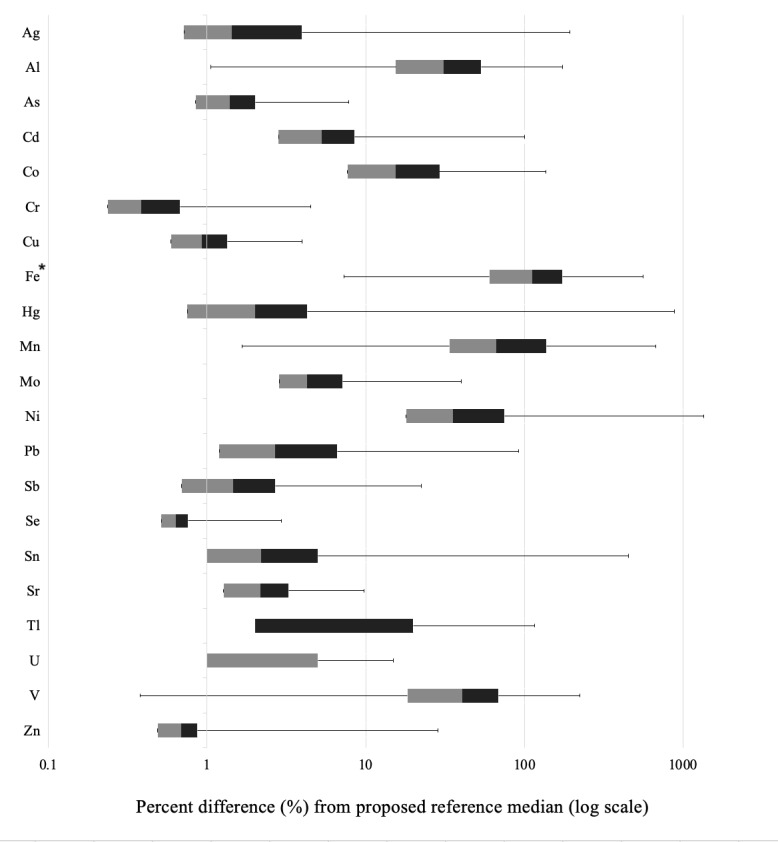
Subject metal concentration (μg/g) variability expressed as a percentage from the reference median [25]. x-axis represented logarithmically. Error bars representing minimum and maximum metal concentration percentage change from reference. *Compared relative to proposed reference maximum described separately by Meramat et al. [26].

The median toenail concentrations of all analysed metals exceeded the proposed reference values for an unexposed population, except for Cr and Se [24,25]. Metal concentrations showed substantial variability among participants, with many individuals exhibiting extremely high metal concentrations (**Figure 1**).

### Peripheral hearing test results

**Figure 2:**
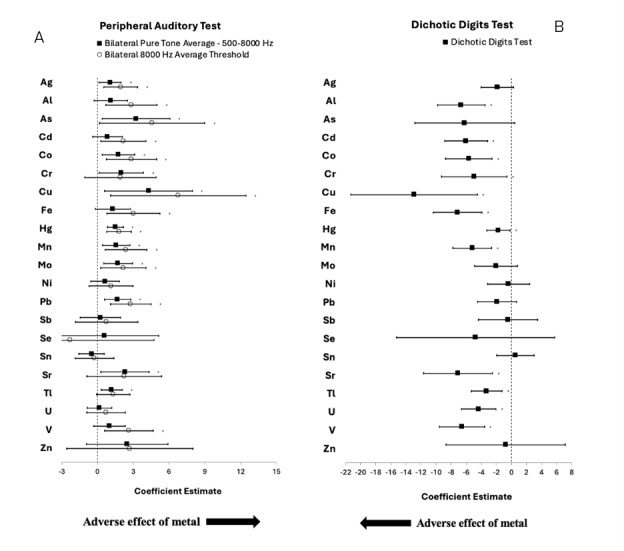
Forest Plot of Coefficient Range for Audiometry Tests. **Panel A.** Peripheral audiometry. **Panel B.** Dichotic Digits Test.

The mean PTA for the group was within the normal range (PTA ± SD = 11 ± 4.0 dB HL (**Table 1**)). Elastic Net regression with bootstrapping identified Ag, Cd, Cr, Hg, and Mo as predictors of PTA performance more than 50% of the time (**Table 2**). At the 8 kHz high frequency threshold, As, Ni, Pb, and Sn were also identified (**Table 2**). After accounting for age, noise exposure, and other covariates, multiple significant correlations were observed between audiometric thresholds (mean binaural PTA) and toenail metal concentrations in the linear regression analysis with FDR. Higher concentrations of metals including Ag, As, Co, Cr, Cu, Hg, Mn, Mo, Pb, Sr, and Tl were associated with worse mean binaural PTA (**Figure 2**, Panel A, **Table 2**). Likewise, elevated hearing thresholds at 8 kHz were found in children with high toenail concentrations of Ag, Al, As, Cd, Co, Cu, Fe, Hg, Mn, Mo, Pb, and V (**Table 2**). Across both Elastic Net and linear regression methods, Ag, Cr, Hg, and Mo consistently emerged as predictors of PTA performance. At the 8 kHz high-frequency threshold, both analyses also identified As and Pb as associated metals.

**Table 2:** Multiple linear regression *P*-values *vs*. Elastic Net percent predictor results for central auditory processing and peripheral auditory tests*

	Binaural PTA		8000 Hz		Dichotic Digits Test	
**Metals**	**% predictor**	***P-*value**	**% predictor**	***P-*value**	**% predictor**	***P*-value**
Silver	53.1	0.02	-	0.01	-	-
Aluminum	-	-	-	0.01	-	≤0.001
Arsenic	-	0.02	61.6	<0.05	-	-
Cadmium	61.2	-	-	0.03	-	≤0.001
Cobalt	-	0.02	-	0.01	-	≤0.001
Chromium	61.5	0.03	-	-	-	0.02
Copper	-	0.02	-	0.02	-	≤0.003
Iron	-	-	-	0.01	-	≤0.001
Mercury	57.8	≤0.001	-	<0.001	-	0.02
Manganese	-	0.01	-	0.01	-	<0.001
Molybdenum	59.3	0.01	-	0.04	-	-
Nickel	-	-	53.5	-	-	-
Lead	-	0.00	56.8	<0.002	63.3	-
Antimony	-	-	-	-	60.3	-
Selenium	-	-	-	-	-	-
Tin	-	-	54	-	-	-
Strontium	-	0.03	-	-	-	≤0.003
Thallium	-	0.01	-	-	-	≤0.003
Uranium	-	-	-	-	79.1	≤0.001
Vanadium	-	-	-	0.01	52.8	≤0.001
Zinc	-	-	-	-	-	-

ASGM – Artisanal Small Scale Gold Mining, DDT – Dichotic Digits Test, PTA – Pure Tone Audiometry

*P < 0.05 and satisfying False Discovery Rate <0.2. Feature selection in Elastic Net >50%

### Central Auditory Processing test results

In the Elastic Net regression with bootstrapping, Pb, Sb, U, and V were identified as predictors of DDT performance more than 50% of the time (**Table 2**). After accounting for age, noise exposure, and other covariates, elevated toenail concentrations of Al, Cd, Co, Cr, Cu, Fe, Hg, Mn, Sr, Tl, U, V, and Zn were associated with poorer performance (lower percentage of correct responses) on the DDT (**Figure 2**, Panel B **Table 2**). Taken together, both tests identified U and V as potential predictors of central auditory processing.

### Covariate analysis

The inclusion of 14 different covariates from the questionnaire data resulted in relatively minor changes in *P*-values. Multiple covariates affected the results for As and the mean binaural 8 kHz threshold, including sex, other toxic exposures (gentamicin, amikacin, pesticides, and cyanide), sum of hours in mining activities, memory impairment, frequent headaches, and frequent dizziness. The relationship with As and binaural PTA was also affected by sex and the sum of hours in mining activities. Sex also affected the relationship with Cr and binaural PTA. The sum of hours in mining activities affected the relationship between Cu and binaural PTA. The relationship between Sr and binaural PTA was also affected by reported mercury handling in the mines. Finally, the relationship with Hg and DDT was affected by a history of both otalgia and frequent headaches. This covariate effects are generally associated with weak statistical correlations with *P*-values that are near the 0.05 cut-off and the effect of multiple analyses.

## DISCUSSION

### Overview

This study yielded three significant findings. First, children and adolescents in ASGM communities had exceedingly high levels of multiple potentially toxic heavy metals. Second, hearing thresholds and CAP test performance, specifically the DDT of binaural integration, were associated with heavy metal concentrations in toenails. These relationships were independent of age and noise exposure, even among children with normal hearing thresholds. Finally, minimally trained CHWs could detect these changes using a tablet-based system in remote ASGM communities. These findings reveal metals with previously under-recognised neurotoxic potential, underscoring the need for further research into the underlying mechanisms of auditory pathway vulnerability.

Previous research on the health effects of those living in ASGM communities has primarily focused on Hg toxicity. This large-scale comprehensive study confirms previous findings that residents of ASGM communities are exposed to high levels of multiple other heavy metals aside from Hg [6]. With the exception of Cr and Se, median toenail concentrations far exceeded proposed reference values for all tested metals in children and adolescents. Considerable variability, particularly at the upper limits, was observed between participants, which could be due to differences in exposure timing, metabolic variations, or complex interactions between metals. The children and adolescents also reported a high rate of neurological symptoms including memory loss, dizziness, balance problems, tremor, and paraesthesia, possibly due to the combined effects of multiple toxic heavy metals.

While some heavy metals have been proposed to cause ototoxicity, few studies have investigated behavioural CAP testing in relation to heavy metal toxicity, and none have studied multiple metals [8]. In our study, both Elastic Net and linear regression identified Ag, As, Cr, Hg, Mo, and Pb as potential predictors of peripheral auditory function. CAP deficits were seen for U and V in both statistical analyses. No associations were found in either analysis between metal concentration and any audiometric test for Se and Zn. These findings highlight the complexity of heavy metal exposure's effects on auditory function, emphasising the need for further research and longitudinal studies.

### Mercury

Collectively, heavy metal or metalloid exposure may disrupt auditory processing through various mechanisms including oxidative stress, alteration of neural conduction, and induction of inflammatory cascades, though further investigation is warranted to explore metal-specific effects [26–29]. Of the 21 metals studied, ototoxic effects have been reported for Hg, Pb, As, Mn, and Cd. Acute and long-term Hg exposure can cause irreversible damage to both the peripheral and central auditory nervous system [6,7,30]. Dutra, Monteiro, and Câmara found mercury-exposed adolescents had worse performance compared to unexposed children on a non-verbal sound sequence memory test, frequency pattern test, duration pattern test, and dichotic word test despite having normal hearing [30]. Murata et al. [31] reported prolonged auditory brainstem response (ABR) latencies in normal-hearing children with developmental exposure to methylmercury. Our study identified Hg as a predictor of peripheral auditory effects using Elastic Net regression. In contrast, when adjusting for age, noise exposure, and other covariates, linear regression analysis revealed that Hg levels were associated with deficits in both peripheral and central auditory processing.

### Lead

Lead (Pb) exposure has been linked to elevated hearing thresholds and longer ABR latencies in some studies [7]. In a study of cognitive function in Cincinnati school children, blood Pb levels were associated with small, but detectable deficits in degraded speech perception [32], but other studies have had conflicting results. In our study, Elastic Net analysis identified Pb as a predictor of performance at high frequency thresholds (8kHz) and on the DDT. Comparatively, linear regression analysis found that Pb levels were associated with poor peripheral hearing (PTA and 8 kHz).

### Arsenic

Arsenic (As) was linked to changes in peripheral hearing (PTA and 8 kHz), though the high frequency changes were not significant after adjusting for covariates, and no association with CAP was found. Linear regression analysis showed associations between Mn and both peripheral (PTA and 8kHz) and CAP (DDT) effects, though these associations were not observed in Elastic Net with bootstrapping. Lastly, Cd was associated with changes in DDT and high-frequency thresholds, while Elastic Net with bootstrapping identified it as a potential predictor of PTA.

### Novel metal ototoxins

There were several metals identified in both analyses which have not previously been known to induce ototoxicity. The relationship between Ag, Mo, U, and V and peripheral or central auditory function is not well-established. These metals have been implicated in neurotoxicity to varying degrees, including potential cognitive effects. Our findings suggest that these less studied metals should be included in future studies to improve our understanding of their potential ototoxic or neurotoxic effects.

### Implications for global health

Currently, few global initiatives have implemented targeted screening and remediation efforts, but large-scale, population-wide surveillance remains largely limited across LMICs due to infrastructure constraints and the high cost of biomonitoring. This is the first study to use CHW’s for audiometric evaluations in remote ASGM communities and the first to use CHW’s for CAP assessments in these settings. This approach expands the role of CHWs in screening for hearing pathologies and delivering findings to specialists for management. The employment of CHWs to support culturally appropriate outreach helps to build trust with ongoing engagement and education remaining essential for long-term success. CHWs-led testing could enable improved longitudinal monitoring, enhancing health care delivery and our understanding of the long-term impact of these toxins on the nervous system.

### Limitations

This study has several limitations. These results cannot be broadly generalised other LMIC populations and it is also important to consider alternate sources of heavy metal exposure as seen in consumer products, industrial emissions, and contamination of food and water [33]. The degree and variety of metal exposure is similar to that reported by our team and others for ASGM communities. However, these findings are not intended to be representative of high-risk populations from other occupational exposures. Additionally, toenail samples can be susceptible to contamination from the environment, which could give falsely positive results. The Trace Elements Analysis Laboratory at Dartmouth College, however, has rigorous protocols to limit the effect of contamination. The inability to address noise exposure comprehensively is a major limitation, as self-reported exposure is subject to recall bias, and noise intensity was not measured. Although participants were classified using categorical exposure groups derived from survey responses, this approach may not fully capture the variability or intensity of true noise exposure. Elastic Net with bootstrapping may also have mitigated this issue, as each bootstrap sample likely included a different composition of individuals with significant noise exposure in each iteration. Future studies might prospectively employ dosimeters to evaluate noise exposures more accurately. Additionally, this study did not fully explore potential synergistic effects of multiple metal exposures. Though covariate risk factors did not significantly alter the results in the linear regression analyses, the findings could be influenced by other overlapping risk factors not included in this analysis. The aetiology of hearing loss and other neurologic conditions in this population may involve a complex interaction of multiple risk factors, with toxic heavy metal exposure being one of many potential causes. Nevertheless, the persistence of these associations seen when specific covariates were included and after multiple bootstrapped samples, suggests that these associations are not merely the result of confounding variables. Finally, the Elastic Net statistical method employed here requires additional validation with independent cohorts. Further research is needed to better understand the contributions of all aetiologies to hearing loss and CAP deficits in these communities.

## CONCLUSIONS

This study successfully used CHWs to assess both hearing thresholds and CAP in a remote ASGM setting. The children in this study had exceedingly high levels of numerous heavy metals with substantial variability in the pattern and degree of exposure. Although mercury is often considered the primary metal exposure in ASGM, this study found it was not the only significant heavy metal exposure. There were several correlations between the concentrations of multiple heavy metals and audiometric testing. These correlations included heavy metals known for their ototoxic and neurotoxic effects (Hg, Pb, and As), as well as other metals (Mo, Ag, Cr, U, and V) that are not commonly linked to these effects. This exploratory analysis uncovers associations that merit further investigation. Additional studies are needed to understand the pathways through which these metals exert their toxicity.

## Additional material


Online Supplementary Document
